# Polydopamine and Mercapto Functionalized 3D Carbon Nano-Material Hybrids Synergistically Modifying Aramid Fibers for Adhesion Improvement

**DOI:** 10.3390/polym14193988

**Published:** 2022-09-23

**Authors:** Zhonghang Fang, Qunzhang Tu, Xuan Yang, Xinmin Shen, Qin Yin, Zhiyuan Chen

**Affiliations:** 1 College of Field Engineering, Army Engineering University of PLA, Nanjing 210007, China; 2 China Astronaut Research and Training Center, Beijing 100094, China

**Keywords:** aramid fiber, polydopamine, graphene oxide, carbon nanotube, interfacial adhesion

## Abstract

In order to solve the problem of poor interfacial adhesion between aramid fibers and a rubber matrix, an efficient and mild modification method was proposed via polydopamine and mercapto functionalized graphene oxide (GO) and carbon nanotube (CNTs) hybrids synergistically modifying aramid fibers. GO and CNTs were firstly stacked and assembled into unique 3D GO-CNTs hybrids through π-π conjugation. Then, the mercapto functionalization of the assembled 3D GO-CNTs hybrids was realized via the dehydration condensation reaction between the hydroxyls of GO and the silanol groups of coupling agent. Finally, the mercapto functionalized 3D GO-CNTs hybrids were grafted onto the aramid fibers, which were pre-modified by polydopamine through the Michael addition reaction mechanism. The surface morphology and chemical structures of GO-CNTs hybrids and fibers and the interfacial adhesion strength between fibers and rubber matrix were investigated. The results showed that the modification method had brought about great changes in the surface structure of fibers but not generated any damage traces. More importantly, this modification method could improve the interfacial strength by 110.95%, and the reason was not only the reactivity of functional groups but also that the 3D GO-CNTs hybrids with excellent mechanical properties could effectively share interfacial stress. The method proposed in this paper was universal and had the potential to be applied to other high-performance fiber-reinforced composites.

## 1. Introduction

The interface between the reinforcing phase and the matrix phase is a key factor affecting the comprehensive properties of composites [1]. For aramid fiber (AF)-reinforced rubber matrix composite products, such as rubber tracks, conveyor belts and tires, etc., the reinforcing phase (aramid fiber) is a kind of brittle material, while the matrix phase (rubber) shows excellent elasticity. That is to say, when bearing a large load, the reinforcing phase would break before the final failure of the composites. The interfacial performance plays an important role in the mode of crack propagation at the end of fibers, which has a crucial effect on the load transmission. Aramid fiber with the advantages of high strength and light weight has the defects of strong chemical inertia and weak interfacial adhesion with other materials [2,3,4]. Therefore, the improvement of interfacial performance between aramid fiber and rubber is a key and hot issue to be studied.

Currently, the main methods of the surface modification of aramid fiber can be divided into physical and chemical methods. The physical methods mainly include ultrasound [5], high-energy radiation [6,7], plasma treatment [8], UV-initiated grafting [9], etc. The physical modification methods with simple process technologies have the disadvantages of a difficult industrialization process and an unstable modification effect. In comparison, the widely studied chemical methods mainly include acid etching [10], chemical graft [11,12], and enzyme catalysis [13], which can directly introduce active groups onto the fiber surfaces but can easily cause irreversible damages to the fiber structures. Therefore, finding a mild and efficient physical–chemical mixed method to modify the surface of aramid fibers has become the research emphasis.

Some characteristics of natural organisms often become a breakthrough for researchers to solve research problems. Inspired by the mechanism of biological adhesion proteins in marine mussels, polydopamine (PDA), a polymer obtained via the oxidative self-polymerization of mussel adhesion protein molecule-dopamine [14,15], is considered an adhesive material with great application potential and has been gradually applied to the surface modification of high-performance fibers due to its high chemical activity [16,17]. At present, the adhesion mechanism of dopamine is still controversial, but researchers generally believe that catechol and the amino group play a central role [18]. Based on the differences in the surface activity of substrates, PDA can form an adhesive coating on the surface of substrates through covalent or non-covalent interaction [19,20]. In addition, PDA has the advantage of not damaging the substrate structure [21].

Nanomaterials have developed rapidly in the past decade and have been widely used in many fields [22]. As a typical carbon nano material, carbon nanotubes (CNTs) with a one-dimensional (1D) structure have an extremely high tensile strength of 11–63 GPa and a Young’s modulus of 270–950 GPa and have been reported to be a promising high-performance fiber surface modification material due to its effect on the surface structure of fibers and the enhancement of interfacial strength [23]. However, due to the lack of active groups, it is difficult for CNTs to directly interact with inert high-performance fibers and then be grafted on the fiber surface [24]. In comparison, as another typical carbon nano material, graphene oxide (GO) with a two-dimensional (2D) structure not only has excellent mechanical properties but also contains rich oxygen-containing groups, such as hydroxyl and carboxyl groups [25,26]. It can be seen that the synergistic use of CNTs and GO is an ideal solution.

In this paper, we proposed a mild and universal modification method via polydopamine and mercapto functionalized 3D GO-CNTs hybrids synergistically modifying aramid fibers to improve the interfacial adhesion with rubber matrix. Firstly, the 3D GO-CNTs hybrids were assembled under ultrasonic condition via π-stacking interaction formed between the π-conjugated multiple aromatic regions of GO sheets and the sidewalls of CNTs. Then, the GO-CNTs hybrids were mercapto functionalized through the dehydration condensation reaction between hydroxyls of GO and silanol groups of the coupling agent MPTMS. Finally, the mercapto functionalized 3D GO-CNTs hybrids were grafted onto the pretreated aramid fibers with an initial layer of poly-dopamine via the Michael addition reaction. Since mercapto groups can participate in the vulcanization reaction of rubber [27,28], nano materials containing mercapto groups can be grafted onto the surface of AF to achieve covalent bonding between fibers and the rubber matrix, thereby improving the interfacial adhesion. The surface morphology and chemical structures of GO-CNT hybrids and fibers and the interfacial adhesion strength between fibers and rubber were characterized to verify the effectiveness of this modification method.

## 2. Materials and Methods

### 2.1. Materials

The aramid fibers (Kevlar K-29, 3300 dtex, 14–15 μm in average diameter) used in this study were received from Changzhou Gaoyuan Group Co., Ltd., Changzhou, China. Methanol, ethyl acetate, acetone, deionized water, glacial acetic acid, dopamine hydrochloride, tris (hydroxymethyl) aminomethane (Tris), and (3-mercaptopropyl) trimethoxysilane (MPTMS) were purchased from Shanghai Aladdin Industrial Co., Ltd., Shanghai, China. Graphene oxide (GO) and multi-walled carbon nanotubes (CNTs) were provided from Nanjing Xianfeng Nanotechnology Co., Ltd., Nanjing, China. The rubber ingredients were obtained from Nanjing Xinyue Chemical Industrial Co., Ltd., Nanjing, China, and were listed in Table S1. The properties of the aramid fibers, CNT, and GO are shown in Supplementary Materials S1.2.

### 2.2. GO-CNTs Hybrids and PDA Synergistically Modifying the AF Surface

**Figure 1:**
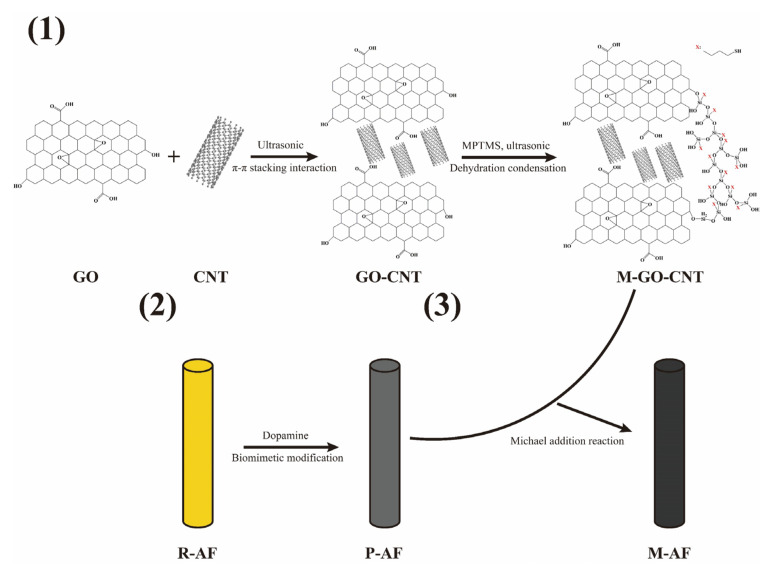
Schematic illustration of the surface modification of aramid fibers.

Figure 1 shows the schematic illustration of AF surface modification, which can be summarized as follows:

(1) GOs (0.5–5 μm in sheet diameter and 0.8–1.2 nm in thickness) and CNTs (8–15 nm in diameter and 0.5–2 μm in length) with a mass ratio of 2:1 were added into deionized water, and the concentration of carbon nano-materials was controlled to 0.5 mg/mL. After stirring for 15 min, the mixture liquor was treated by ultrasound for 6 h to prepare a 3D carbon nano-material hybrid (which were labeled GO-CNTs) suspension. The dried GO-CNTs and a little glacial acetic acid were added into a methanol/water/MPTMS solution (wherein the volume ratio was controlled to 7:2:1) to be treated by ultrasound for 4 h. The mercapto functionalization of GO-CNTs was realized via the dehydration condensation reaction between the hydroxyls of GO and the silanol groups of MPTMS. The reacted nano-materials were labelled M-GO-CNTs.
(2) The AFs were cleaned with ethyl acetate and acetone, then dried and labelled R-AF. The cleaned R-AFs were immersed into a dopamine solution with a concentration of 2 g/L and a pH value of 8.5, followed by stirring at room temperature for 12 h. After the reaction, AFs were rinsed with water to remove the excess reagents, followed by drying at 100 °C for 3 h. The reacted AFs were labelled P-AF.
(3) M-GO-CNTs were added into a methanol/water solution (the volume ratio was 7:2) and then treated by ultrasound for 2 h to prepare a M-GO-CNT suspension. P-AFs were immersed into this suspension, whose pH value of 8.5 was adjusted by tris (hydroxymethyl) aminomethane. The mixture was stirred slowly for 12 h. After the reaction, AFs were rinsed with methanol to remove the excess reagents, followed by drying at 100 °C for 3 h. The reacted AFs were labelled M-AF.

### 2.3. Preparation of AF/Rubber Composites

**Figure 2:**
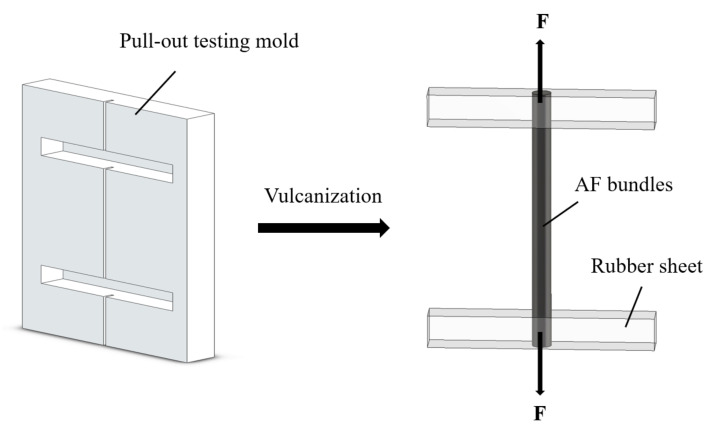
Schematic illustration of the preparation and pull-out test for AF/rubber composites.

The AF/rubber composites were prepared for the pull-out test, which was an effective way to characterize the interfacial adhesion according to GB/T 2942-2009. The rubber matrix for the test were firstly plasticized and mixed, which was detailed in Supplementary Materials S1.1. Then, the rubber matrix and AF were placed in the testing mold, where both ends of the AF bundles were covered with rubber sheets. The size of the groove swhere the rubber sheet and AF bundles were placed in the mold were 50 mm × 6.4 mm × 6.4 mm and 30 mm × 0.8 mm × 3.2 mm, respectively. Finally, The AF/rubber composites were obtained by vulcanization treatment at 140 °C for 20 min under 20 MPa. For the AF/rubber composites for the pull-out test, the gauge length of the AF bundles between rubber sheets and the depth of AF bundles embedded in the rubber sheet were 8.6 mm and 6.4 mm, respectively. Figure 2 shows the schematic illustration of the preparation and pull-out test for AF/rubber composites.

### 2.4. Characterization

The GO-CNTs before and after functionalization were characterized by TEM (FEI Tecnai G2 F20, Waltham, MA, USA), XPS (ESCALAB 250XI, Thermo, Electron Corporation, Waltham, MA, USA), FTIR (PerkinElmer Spectrum Two, Waltham, MA, USA), and XRD (SmartLab 9, Rigaku, Tokyo, Japan) to verify the structures of functionalized 3D carbon nano-material hybrids. The AFs before and after surface modification were characterized by SEM (Hitachi S-4800, Hitachi, Tokyo, Japan), XPS, and FTIR to illustrate the effectiveness and mildness of modification. Details of sample preparation for the above tests are shown in Supplementary Materials S1.3. According to GB/T 2942-2009, the pull-out test was conducted to analyze the interfacial adhesion between AFs and rubber matrix by using universal testing machine (Wance Testing Machine ETM104C, Shenzhen, China). The tensile speed of the machine is 100 mm/min, and the force is applied along the longitudinal axis of the fibers. The average of eight samples at the same conduction was recorded as the final result, to reduce test error.

## 3. Results and Discussion

### 3.1. Characterizations of 3D Carbon Nano-Material Hybrids

**Figure 3:**
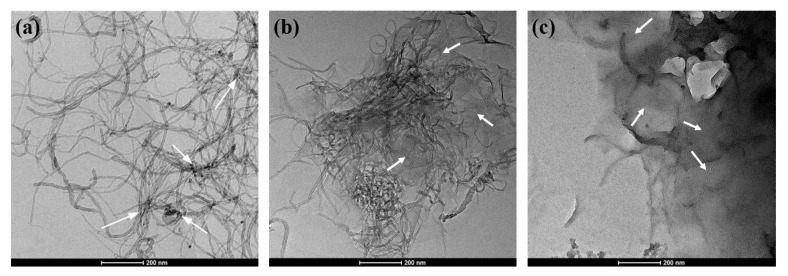
TEM micromorphology of (**a**) CNTs, (**b**) GO-CNTs, and (**c**) M-GO-CNTs.

Figure 3 shows the micro morphology of carbon nano-materials. In Figure 3a, it can be clearly observed that the entanglement between most CNTs is very serious. After treated by GO, the TEM image of GO-CNTs in Figure 3b shows that the entanglement of CNTs tends to decrease more than common pure CNTs [29,30], which indicates that the assembly of GO and CNTs can improve the dispersibility of CNTs. Moreover, some CNTs are inserted into the lamellae of GO, while the rest are distributed at the edge of GO. Comparatively, the TEM image of the M-GO-CNTs in Figure 3c shows a larger shaded area and more stacking GO lamellae, and more CNTs are evenly distributed into the lamellae of GO to form the more stable 3D hybrids, which is related to the bridge role of silane coupling agent MPTMS in the mercapto functionalization treatment process of the hybrids.

**Figure 4:**
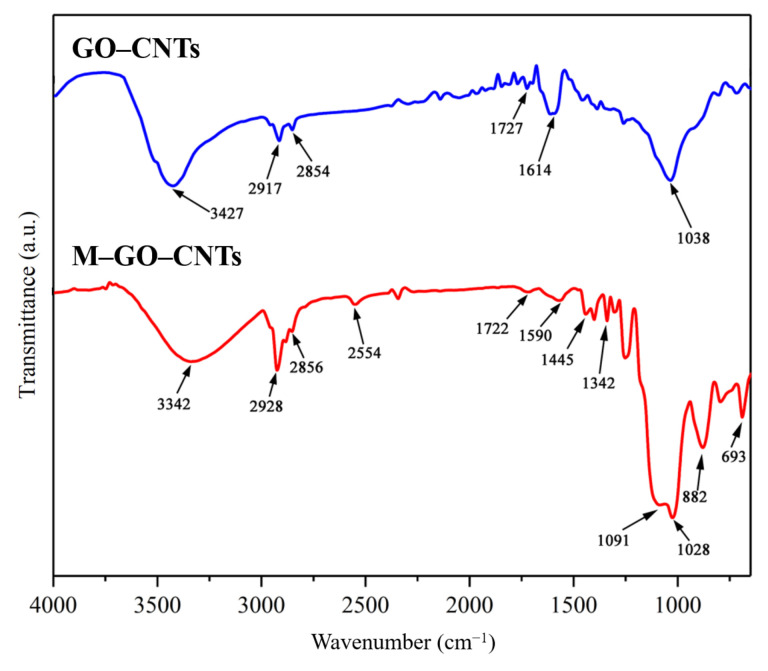
FTIR spectra of GO-CNTs and M-GO-CNTs.

Figure 4 shows the FTIR spectra of GO-CNTs and M-GO-CNTs. The absorption peaks of GO-CNTs are located at 3427 cm^−1^, 2917 cm^−1^, and 2854 cm^−1^ (double peaks), 1727 cm^−1^, 1614 cm^−1^, and 1038 cm^−1^, which are attributed to -OH stretching vibration, methylene stretching vibration, C=O stretching vibration, C=C stretching vibration, and C-O stretching vibration, respectively [31]. It is worth noting that, due to the lack of chemical groups on the surface of CNTs, the absorption peaks of GO-CNTs mainly comes from GO. Compared with the FTIR spectrum of GO-CNTs, there are several new absorption peaks appearing in that of M-GO-CNTs. Thereinto, 2554 cm^−1^, 1342 cm^−1^, and 693 cm^−1^ are related to sulfur, which can be attributed to S-H stretching vibration, S-CH_2_ symmetrical deformation vibration, and S-C stretching vibration, respectively. Moreover, 1445 cm^−1^, 1091 cm^−1^, and 882 cm^−1^ are related to silicon, which can be attributed to CH_2_ shear vibration in Si-CH2, Si-O-Si antisymmetric stretching vibration, and Si-OH stretching vibration [32]. These new peaks indicate the successful realization of mercapto functionalization.

**Figure 5:**
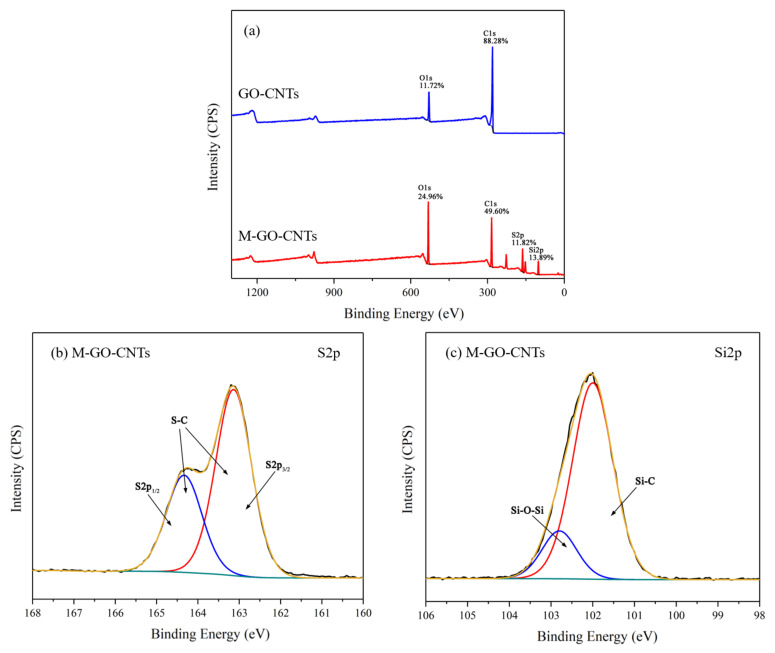
XPS wide-scan spectrum (**a**), S2p (**b**), and Si2p (**c**) high-resolution spectra of M-GO-CNTs.

Figure 5 shows the XPS spectra of GO-CNTs and M-GO-CNTs. It can be deduced from the XPS wide scan spectrum of Figure 5a that GO-CNTs are mainly composed of carbon (C) and oxygen (O), whose contents are respectively 88.28 at.% and 11.72 at.%, wherein oxygen is mainly attributed to the oxygen-containing functional group of GO. By contrast, the new peaks of S2p and Si2p can be observed in the XPS wide scan spectrum of M-GO-CNTs, and the contents of sulfur (S) and silicon (Si) are 11.82 at.% and 13.89 at.%, respectively. Moreover, the O content was increased, while the C content was decreased, obviously. The changes in elements and their content can initially prove the functionalization of the carbon nano-materials. As shown in Figure 5b, for the S2p high-resolution spectrum of M-GO-CNTs, the curve can be fitted to S2p_1/2_ at 164.3 eV and S2p_3/2_ at 163.1 eV, which indicates the existence of an S-C bond [33]. For the Si2p high-resolution spectrum of M-GO-CNTs in Figure 5c, the fitted peaks of 102.0 eV and 102.8 eV can be attributed to Si-C and Si-O-Si, respectively [34].

**Figure 6:**
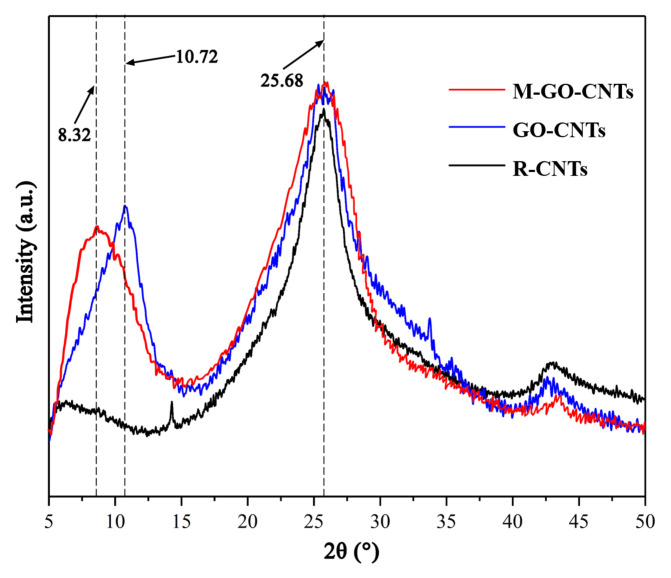
XRD spectra of R-CNTs, GO-CNTs, and M-GO-CNTs.

Figure 6 shows the XRD spectra of R-CNTs, GO-CNTs, and M-GO-CNTs. For R-CNTs, only one strong diffraction peak (002) can be found, located at 2θ = 25.68°, which is derived from free graphite carbon in CNTs. For the XRD spectrum of GO-CNTs, there are two strong diffraction peaks, of which the diffraction peak located at 2θ of 10.72° can be attributed to the oxygen-containing functional groups between the GO lamellar structures, while the other one located at 2θ of 25.68° indicates the assembly between GO and CNTs. After the 3D GO-CNTs hybrids are assembled, the crystal plane spacing is 0.825 nm. Compared with R-GO, it can be found that there are a certain number of CNTs in the hybrid lamellae. After mercapto functionalization, in the XRD spectrum of M-GO-CNTs, the 2θ value corresponding to the strong diffraction peak (001) becomes 8.32°, that is, the crystal plane spacing at this time is significantly increased to 1.063 nm, which is sufficient to illustrate that, for the 3D structure of GO-CNTs, functionalized grafting changes its crystal structure.

### 3.2. Characterizations of Aramid Fibers

**Figure 7:**
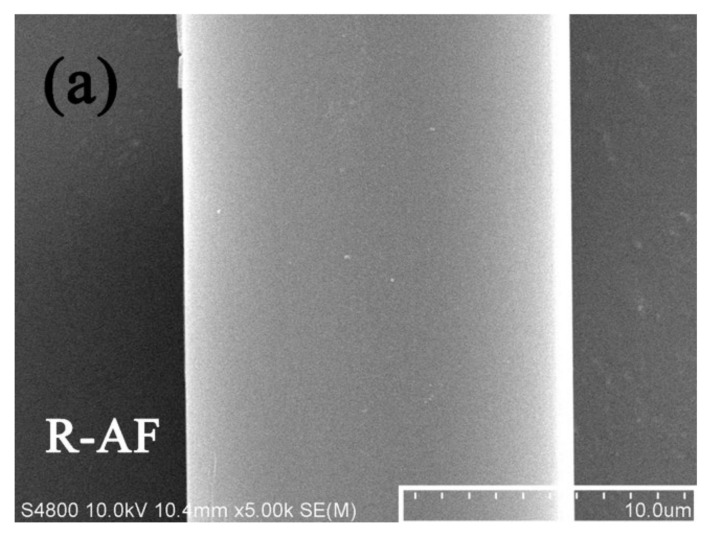

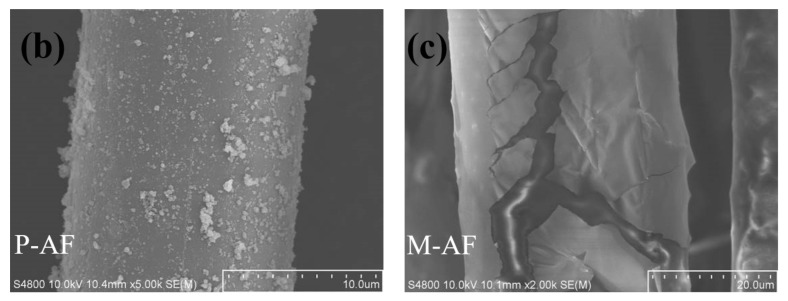
Surface morphologies of (**a**) R-AF, (**b**) P-AF, and (**c**) M-AF.

Figure 7 shows the surface morphologies of AFs before and after surface modification. In the SEM images of the surface morphology as shown in Figure 7a, the surface of R-AF is smooth and almost free of any defects due to high crystallinity. After being treated by the oxidative self-polymerization of dopamine, the PDA particles are observed and relatively evenly distributed on the surface of P-AF (Figure 7b). Furthermore, there is no etching or damage trace observed on the P-AF surface, namely, the pretreatment of fibers does not generate micro structural damage on the surface. As shown in Figure 7c, by contrast, a great deal of sheet-like morphology is observed on the surface of M-AF, and the fiber diameter is increased significantly (from 14.5 μm to 38.9 μm), which indicates the successful graft of 3D carbon nano-material hybrids. Obviously, through the modification treatment, the surface structure of M-AF has changed significantly, and the surface is no longer as smooth as that of R-AF. Moreover, although the graft of 3D carbon nano-material hybrids can improve the diameter of a single-filament fiber to a certain extent, it would not be expected to be so significant. By the wrapping effect of sheet structures, it inferred that the dramatic improvement of fiber diameter may be attributed to the micro feature of the “integrate phenomenon” of several single-filament fibers after surface modification.

**Figure 8:**
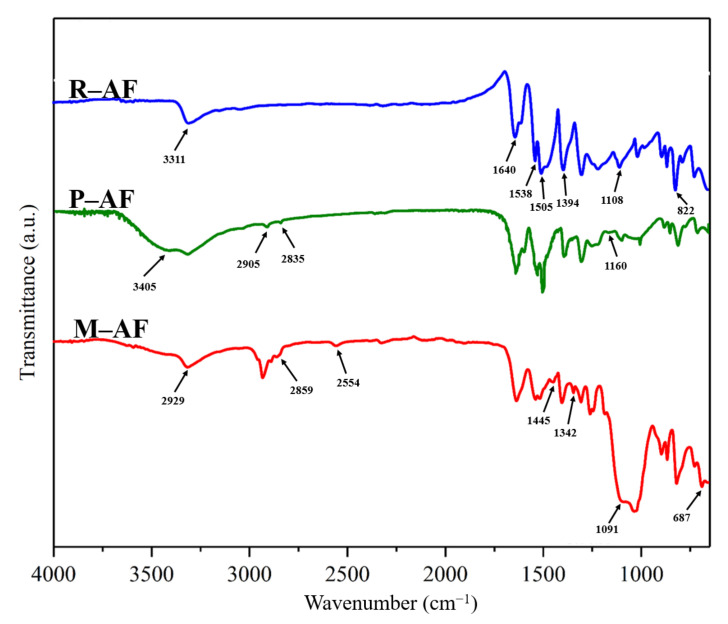
FTIR spectra of R-AF, P-AF, and M-AF.

Figure 8 shows the FTIR spectra of AFs before and after surface modification. For the FTIR spectrum of R-AF, the peaks located at 3311 cm^−1^ and 1538 cm^−1^ are attributed to the stretching vibration and deformation vibration of N-H, respectively, while the C-N stretching vibration generates two peaks at 13941 cm^−1^ and 1108 cm^−1^. The other three peaks, at 1505 cm^−1^, 1640 cm^−1^ and 822 cm^−1^, are respectively ascribed to the stretching vibration of C=C and C=O from amide groups and the deformation vibration of C-H [35]. After treated by PDA, the stretching vibration of -OH and C-O from PDA generates new peaks at 3405 cm^−1^ and 1160 cm^−1^, respectively, while the stretching vibration of the methylene group generates double peaks at 2905 cm^−1^ and 2835 cm^−1^ [36]. By contrast, for the FTIR spectrum of M-AF, it is worth noting that the new peaks at 2554 cm^−1^, 1342 cm^−1^, 687 cm^−1^, 1445 cm^−1^, and 1091 cm^−1^ can be attributed to the S-H stretching vibration, S-CH_2_ symmetrical deformation vibration, S-C stretching vibration, CH_2_ scissoring vibration in Si-CH_2_, and Si-O-Si antisymmetric stretching vibration, respectively. In short, the result of FTIR can fully confirm the successful graft of functionalized 3D carbon nano-materials on the surface of aramid fibers.

**Figure 9:**
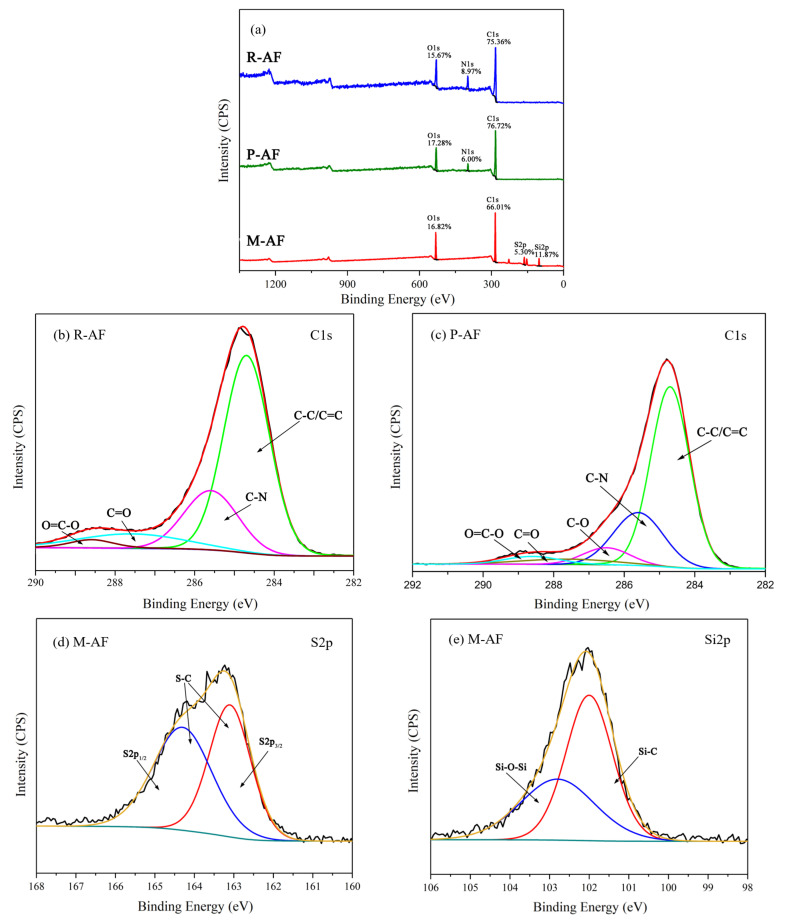
XPS wide-scan spectrum (**a**), C1s high-resolution spectra of (**b**) R-FA and (**c**) P-AF, S2p (**d**) and Si2p, and the (**e**) high-resolution spectra of M-AF.

Figure 9 shows the XPS spectra of AFs before and after surface modification. As shown in Figure 9a, the surface of R-AF and P-AF are mainly composed of C, N, and O, and there is no significant change in content, while the new peaks related to Si and S appear in the XPS wide-scan spectrum of M-AF, which can be ascribed to the graft of mercapto groups. Meanwhile, the N1s peak disappeared, showing that the thickness of film is thicker than the detecting depth of the probe. In Figure 9b, the C1s high-resolution spectrum of R-AF can be fitted into four peaks, including the C-C/C=C peak at 284.6 eV, the C-N peak at 285.5 eV, the C=O peak at 287.5 eV, and the O=C-O peak at 288.5 eV [37]. In contrast, for the C1s high-resolution spectrum of P-AF in Figure 9c, a new peak, C-O at 286.4 eV, can be observed, which can be attributed to the phenolic hydroxyl group from PDA [38]. Based on the treatment of mercapto functionalization, the S2p and Si2p new peaks of M-AF were fitted, and the results are shown in Figure 9d,e, respectively. The S2p high-resolution spectrum of M-AF can be fitted to S2p_1/2_ at 164.3 eV and S2p_3/2_ at 163.1 eV, indicating the existence of an S-C bond, while the fitting peaks at 102.0 eV and 102.8 eV in the Si2p high-resolution spectrum were attributed to Si-C and Si-O-Si, respectively. This result is consistent with the FTIR spectrum.

### 3.3. Interfacial Adhesion Property of AF/Rubber Composites

The interfacial adhesion between AFs and the rubber matrix was evaluated by a pull-out test, and the result is shown in Table 1. Under the same test conditions such as the same gauge length and embedded depth, the same testing mold and environment, the maximum pull-out forces of R-AF and M-AF bundles with rubber were 28.3 ± 1.8 N, 38.07 ± 2.46 N and 59.7 ± 4.7 N, respectively. Due to the smooth surface and lack of active groups, it can be found that the pull-out force between R-AF and the rubber is very low. After being treated by PDA, the appearance of PDA particles improves the surface roughness of the fiber to a certain extent. In addition, since PDA is rich in a variety of active groups, this improves the interfacial molecular interaction between fibers and the rubber. These factors may be used to explain the reason for the increase of pull-out force between P-AF and the rubber after R-AF is treated by PDA. However, there are no groups on the surface of PDA that can directly participate in the vulcanization reaction of rubber, which limits the improvement of the interfacial adhesion strength of the composites to a certain extent [39,40]. For M-AF, it can be found that the interfacial strength is increased by 110.95% after the surface modification, showing the excellent interfacial adhesion. Obviously, the increased roughness generated by the graft of nano-materials is one of the reasons for the improvement of the interfacial performance. Furthermore, the functionalized 3D carbon nano-material hybrids with abundant mercapto groups can also promote the co-vulcanization reaction between AFs and the rubber.

**Figure 10:**
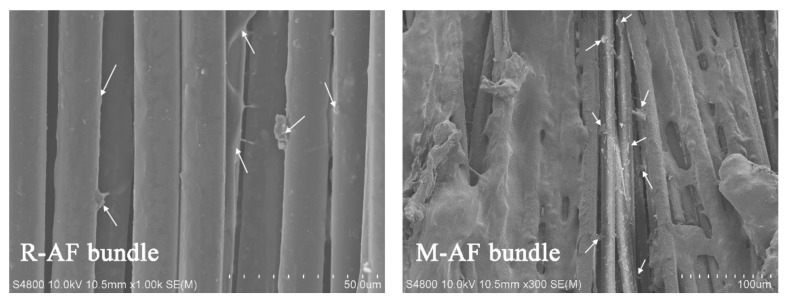
Interlaminar fracture surface morphology after a pull-out test.

Figure 10 shows the interlaminar fracture surface morphology of the composites after the pull-out test. There is a little residual rubber remaining on the surface of the R-AF bundle (as shown by arrows), while a large amount of residual rubber remains on the surface of the M-AF bundle, which indirectly proves that the modified method can effectively improve the interfacial adhesion performance. The change in the residual rubber content on the fiber surface indicates that the fracture of the composite is transferred from the interface region to the rubber side. Most importantly, the conclusion that 3D carbon nano-material hybrids can share the interfacial stress and play a traction role between the reinforcing phase and the matrix phase can be obtained from the micro morphology of the fiber bundle after pull-out tests.

## 4. Conclusions

In this work, in order to improve the interfacial adhesion between aramid fibers and the rubber matrix, an efficient and mild modification method was proposed by polydopamine and mercapto functionalized 3D graphene oxide and carbon nanotube hybrids synergistically modifying the aramid fiber surface. SEM, TEM, FTIR, XPS, and XRD were conducted to indicate the deposition of 3D hybrids on the fiber surface and the realization of mercapto functionalization, thereby verifying that the proposed modification method was feasible and effective. The results of the pull-out test showed that the pull-out force between fiber bundles and the rubber matrix was increased by 110.95% after the fiber surface modification, presenting a significant improvement in interfacial adhesion. This was not only attributed to the increase of fiber surface roughness and the reactivity of mercapto functional groups but was also related to the grafted 3D carbon nano-materials could effectively share the interfacial stress and play a traction role in interface. In addition, the surface modification method proposed in this study has certain universality and is considered to have the potential to be applied to other kinds of high-performance fiber-reinforced composites.

## Figures and Tables

**Table 1:** The result of the pull-out test for AF/rubber composites.

Samples	Pull-Out Force (N)
R-AF	28.3 ± 1.8
P-AF	38.07 ± 2.46
M-AF	59.7 ± 4.7

## Data Availability

The data that support the findings of this study are available from the corresponding author upon reasonable request.

## Supplementary Materials

The following supporting information can be downloaded at https://www.mdpi.com/article/10.3390/polym14193988/s1, Supplementary Materials S1.1: Plasticizing and mixing process of rubber; Table S1: Rubber ingredients; Supplementary Materials S1.2: Physical and chemical properties of the main materials; Supplementary Materials S1.3: Details of sample preparation for the tests.
