# Supplementary material for: Polydopamine and Mercapto Functionalized 3D Carbon Nano-Material Hybrids Synergistically Modifying Aramid Fibers for Adhesion Improvement
Source: Polymers (Basel). 2022 Sep 23;14(19):3988. doi: 10.3390/polym14193988 (PMC9572269; doi:10.3390/polym14193988)
Supplement: Supplementary file 1 [file polymers-14-03988-s001.zip › polymers-1910614-supplementary.pdf]

# Supplementary Materials

## Polydopamine and Mercapto Functionalized 3D Carbon Nano-Material Hybrids Synergistically Modifying Aramid Fibers for Adhesion Improvement

### 1.1 Plasticizing and mixing process of rubber

The rubber ingredients are shown in **Table S1**. The first stage is the rubber plastication process. 40 phr natural rubbers and 60 phr styrene-butadiene rubbers were firstly plasticized at room temperature by the open mill. The rubber needs to be parked for 24 hours after plastication process. The second stage is the rubber mixing process. The rubber ingredients were mixed in internal mixer at 80°C. the ingredients were added as follows: firstly, styrene-butadiene rubber and natural rubber were mixed in internal mixer for 1 min; then, antioxidant (4010NA), zinc oxide, stearic acid, coumarone indene resin, rubber adhesive (RS) and accelerant (CZ) were mixed for 5 min; afterwards, carbon black, white carbon black and aromatic oil were added to mix for 3 min; finally, rubber adhesive (RA) and sulphur were mixed for1 min.

Table S1 Rubber ingredients.

| Rubber ingredients | Parts per hundreds of rubbers (phr) |
|---|---|
| Natural rubber | 40 |
| Styrene-butadiene rubber | 60 |
| Antioxidant (4010NA) | 1.5 |
| Carbon black | 20 |
| White carbon black | 15 |
| Zinc oxide | 5 |
| Stearic acid | 2.5 |
| Aromatic oil | 10 |
| Coumarone indene resin | 10 |
| Rubber adhesive (RA) | 1 |
| Rubber adhesive (RS) | 1 |
| Accelerant (CZ) | 5 |
| Sulphur | 1 |
| **Total** | 172 |

### 1.2 Physical and chemical properties of the main materials

The type of the aramid fibers in this study is poly-p-phenylene terephthalamide. The analytical chain of the fiber is arranged by the benzene ring and amide group according to certain rules, and the amide group is located at the opposite position of the benzene ring, so this polymer has good

regularity, resulting in high crystallinity of aramid fiber. Therefore, the surface of aramid fiber lacks active groups and is chemically inert. Table S2 shows main properties of aramid fibers. Table S3 and Table S4 list the properties of carbon nanotubes and graphene oxide, respectively. Graphene oxide contains rich oxygen-containing groups, such as hydroxyl and carboxyl groups, while carbon nanotubes is chemically inert.

Table S2 Main properties of aramid fibers

| Property | Value |
|---|---|
| Density/g·cm$^{-3}$ | 1.44 |
| Linear density/detx | 3300 |
| Tensile strength/GPa | 3.38 |
| Elastic strength/GPa | 83 |
| Elongation/% | 3.3 |

Table S3 Properties of carbon nanotubes (CNTs)

| Property | Value |
|---|---|
| Density/g·cm$^{-3}$ | 2.1 |
| Diameter/nm | 8-15 |
| Length/μm | 0.5-2 |
| Purity/% | ＞95% |

Table S4 Properties of graphene oxide (GO)

| Property | Value |
|---|---|
| Areal density/mg·m$^{-2}$ | 0.77 |
| Slice diameter/μm | 0.5-5 |
| Thickness/nm | 0.8-1.2 |
| Purity/% | ＞99% |

### 1.3 Details of sample preparation for the tests

For FTIR test, carbon nano-materials samples (CNTS and GO) were obtained by potassium bromide compression method, where nano material powder and potassium bromide powder were mixed evenly and dried, while fiber samples did not need any pretreatment and were directly tested in ATR mode. For TEM test, nano-materials were first dispersed in ethanol by ultrasonic treatment, and then dropped onto a copper mesh and tested after evaporation of ethanol. For SEM test, the samples need to be pasted on the conductive adhesive and then spattered with gold. For XPS and XRD tests, the samples did not need any pretreatment, where fiber and nano material powder were pasted on the glass slide according to the actual test needs.
